# Evaluation of Carcinogenic Polyaromatic Hydrocarbon Levels in Airborne Particulates Associated with Long-Term Exposure throughout the COVID-19 Pandemic in Makkah, Saudi Arabia

**DOI:** 10.3390/ijerph182312745

**Published:** 2021-12-03

**Authors:** Heba Mohamed Adly, Saleh Ahmed K. Saleh

**Affiliations:** 1 Community Medicine and Pilgrims Healthcare Department, Faculty of Medicine, Umm Al-Qura University, Makkah 21955, Saudi Arabia; hebamadly@hotmail.com; 2 Biochemistry Department, Faculty of Medicine, Umm Al-Qura University, Makkah 21955, Saudi Arabia; 3 Oncology Diagnostic Unit, Faculty of Medicine, Ain Shams University, Cairo 11435, Egypt

**Keywords:** polyaromatic hydrocarbons, carcinogenic compounds, air pollution, air quality, PM10, Makkah, Saudi Arabia, pandemic, COVID-19

## Abstract

Background: The effect of polyaromatic hydrocarbons (PAHs) on human health differs depending on the duration and exposure path. Objective: This study aimed to examine the effects of PAHs on the human health risks associated with long-term exposure both before and throughout the COVID-19 pandemic. Methodology: PM10 sampling for 24 h was conducted at six sampling sites (Al-Haram, Aziziyah, Al Nuzhah, Muzdalifah, Arafat, and Al Awali). On-site measurements were conducted from March 2020 to February 2021. PAHs were analyzed using Perkin Elmer GC/MS, which was adjusted with standard reagents for identifying 16 PAH mixtures. Results: The 24 h average PM10 concentration showed considerable inconsistencies, exceeding the WHO standards used for median exposure (25.0 µgm^−3^). The PAH intensities fluctuated from 7.67 to 34.7 ng/m^3^ in a suburban area, near a rush-hour traffic road, and from 6.34 to 37.4 ng/m^3^ close to business and light manufacturing areas. The highest carcinogenic compound levels were found in the Al-Azizia, Al Muzdalifah, and Al Nuzah areas because of the high traffic density, and the lowest concentrations were found in the Al-Haram and Arafat areas throughout the year, as a result of the COVID-19 pandemic health precautions that were undertaken by the government of Saudi Arabia involving border entry limits and limitations of the Umrah and Hajj seasons. Conclusion: This study period is considered extraordinary as the Saudi Arabian government has undertaken successful preventive measures that have had a great effect both on the spread of the pandemic and in reducing air pollution in Makkah. More studies are required to examine PAHs’ carcinogenic effects after the pandemic measures are eased across Makkah.

## 1. Introduction

Polyaromatic hydrocarbons (PAHs) have been revealed as one of the main contaminated airborne components, with several of their elements having been identified as carcinogenic, mutagenic, and allergenic mediators to human wellbeing [1]. Various findings have evaluated PAH concentration and dispersal in a deferred particulate matter [2]. The segmentation of PAH complexes among the particulate matter and vaporous levels differs within the atmospheric environments, the vapor types, the relations with the mixture and vapor, and the general performance of the mixture in the air [3]. 

Particulate matter with an aerodynamic diameter <10 m (PM10) includes a dense mix of a substantial number of compounds; lots of these compounds are poisonous or carcinogenic. Amongst these are the carcinogenic polycyclic aromatic hydrocarbons (c-PAHs; benzo[b]fluoranthene; B[k]F B[a]A, benzo[a]anthracene; CHRY, chrysene; B[b]F, benzo[k]fluoranthene; B[a]P, indeno[1,2,3-cd]pyrene, benzo[a]pyrene; DB[a,h]A, dibenzo[a,h] anthracene; B[g,h,l]P, benzo[g,h,l]perylene; I[c,d]P), which have been examined in some epidemiologic studies of people exposed to ecological contamination [4,5]. 

The carcinogenic polycyclic aromatic hydrocarbons are an abundant set of several hundred chemically associated compounds, which are environmentally enduring with different forms and diverse levels of harmfulness. They have a harmful impact on the body through a variety of behaviors. PAHs are released into the environment through several paths and are generally noticed as encompassing some of these compounds. Moreover, some PAHs are produced by manufacturing. PAHs’ toxicity effects and their routes were studied by interfering with cellular membranes and enzyme systems. It has been demonstrated that PAHs can cause carcinogenic and mutagenic consequences and are efficient immune suppressants [6,7].

Consequently, PAHs are usually found in the air, soil, and water. Meanwhile, they are very soluble in organic diluters as these compounds are extremely lipotropic. Additionally, PAHs demonstrate a variety of purposes, such as light sensitivity, temperature resistance, conductivity, discharge capability, erosion endurance, and biological action [8]. PAHs have exceptionally distinctive UV transmission density ranges. Every single ring-up configuration has a distinctive UV band; hence, every isomer has a unique UV absorbance band. This is particularly effective in PAH classification. Most PAHs additionally have luminous features, generating distinct wavelengths of light after they are excited by light absorption. The major origin of PAHs is the subjective burst of organic material. Some of these compounds are mainly utilized at the same time as intermediates in medications, farming goods, graphic manufactured goods, thermoset plastics, and loosening raw ingredients with other biochemical production [9,10,11].

### 1.1. Different Sources of Polyaromatic Hydrocarbons

The principal PAH sources in the environment consist of three types: pyrogenic, petrogenic, and biological. Pyrogenic PAHs are produced when natural materials remain subject to high temperatures in minimal oxygen concentration [12]. The devastating extraction and thermal splitting of oil remainders into simpler hydrocarbons are pyrolytic practices that occur deliberately [13]. In the meantime, other inadvertent procedures arise throughout the partial oil incineration in vans, imperfect burning in woodland, and imperfect ignition of gas oils in central heating systems. The pyrogenic procedures varied from about 350 to 1200 °C. Pyrogenic PAHs were commonly observed with greater intensities within metropolitan zones, as well as appearing in places near the main causes of PAHs. Moreover, PAHs can be shaped at low temperatures [14]. It is worth stating that rudimentary oils include PAHs that are produced over the course of several years at temperatures of 100–150 °C. It remains well recognized that PAHs can be produced throughout the inadequate ignition of raw elements. PAHs have correspondingly originated from fossil fuel materials. Furthermore, it has not been confirmed that PAH layoff is formed naturally [15]. For instance, they may be produced through specific shrubs and microorganisms or created throughout the destruction of plants. The creation of PAH types can occur from any natural or anthropogenetic sources [15]. It essentially happens in the process of revealing that imperfect ignition either in nature or in anthropogenic derivatives is the biggest provider of PAHs to the natural ecosystem [16].

### 1.2. PAH Distribution in Environment 

The human-caused sources of PAHs are car exhausts, farming, power stations, industrial coke industries, steel manufacturing, factories, and other manufacturing suppliers [17]. PAHs can be found in larger intensities in municipal settings rather than in countryside locations, as high PAH-producing sources are situated in or near metropolitan regions. When distributed into the air, PAHs are observed in two distinct segments, a mist form and a consistent form in which some PAHs are found on airborne particles [18]. Aquaphobic biological substances, including low vapor pressures such as PAHs, are dispersed into air particulate matter in addition to other compounds, including greater aerosol burdens such as benzene [19]. The inconsistency in the air pressure of various PAH complexes affects specific PAHs headed for circulation into several gaseous phases [20]. 

PAHs with low vapor pressures (benzo(a)pyrene) will be more likely to be dispersed into particles, while PAHs with higher vapor pressures (naphthalene) tend to be related to the vapor phase. Consequently, the comparative circulation of PAHs in the two segments will not be the same in an air sample [21,22]. 

### 1.3. PAHs and Human Exposure

The most important path of PAH exposure in the overall population is inhaling ambient air, consuming a diet that includes PAHs, smoking cigarettes, or breathing in smoke from open firesides [23]. Tobacco smoke includes PAHs, such as benzo(a)pyrene, as well as another 40 common or presumed individual carcinogens [24]. Various yields, including wheat berry, can generate PAHs or soak them up through water, air, or soil. Water also includes PAH quantities since these compounds may be leached from the soil into water, or they can run into water from manufacturing overflows and aquatic unintended spillages throughout oil consignment. Additionally, soil includes PAHs, mainly from airborne effects [25]. Consequently, PAHs consistently arise in the majority of people. Paths of exposure consist of digestion and breathing, in addition to epidermal contact, in work-related and non-occupational situations, equally. Occupational disclosure can similarly occur through the mechanism of people who inhale discharge gases, such as mechanics, drivers, and those involved in the manual labor force in drilling, steelworks, or oil refinement. Certain displays can include more than one path concurrently, influencing the overall immersed dosage, as dermal and inhalation exposures from polluted air [26]. Individuals can be exposed to PAHs in the air and in superficial earth through breathing, digestion, or epidermal contact [27].

### 1.4. PAHs and Human Health

PAHs have been classified as of urgent interest regarding probable exposure and harmful health impacts. PAHs have a broadly distributed dispersion and toxicological significance, but their harmful health effects are not equally understood. The International Agency for Research on Cancer [28] categorizes some PAHs as known to be possibly or probably carcinogenic to humans (Group 1, 2A or 2B). Amongst these are benzo[a]pyrene (Group 1), naphthalene, benzo[b]fluoranthene, chrysene, benzo[k]fluoranthene, and benz[a]anthracene (Group 2B) [28]. Several PAHs are very well known as carcinogens that consequently cause a severe hazard to health welfare. The greatest substantial wellbeing risk likely to occur on or after breathing in PAHs is lung cancer, which has a high-risk probability [29].

The PAH effects in humans differ mostly in the duration and exposure path. Moreover, health condition, age, and lifestyle are important factors when considering PAH effects on humans. PAHs’ short-term health effects are still not clear [23].

Eye inflammation, nausea, and diarrhea are common symptoms resulting from high PAH concentrations during occupational exposures [30]. Nonetheless, it is unknown which elements among the polyaromatic combination are liable for particular health effects. Additionally, some PAHs are assumed to trigger skin rashes and soreness. Naphthalene, benzo(a)pyrene, and anthracene are direct causes of skin problems. However, benzo(a)pyrene and anthracene are also known to be skin sensitizers and the reason for a sensitized effect in both animal and human skin [31].

The health impacts of long-term or persistent exposure to PAHs might involve reduced immune function, cataracts, kidney and liver damage, inhalation difficulties, asthma indications, and lung function malformations [32]. Naphthalene may affect red blood cell breakdown if breathed in or absorbed in large quantities [33]. If a person remains exposed to PAHs, the damaging impacts that might take place mostly differ due to the direction of contact [34].

### 1.5. Hydrocarbon Carcinogenicity

Though unabsorbed PAHs may have toxic impacts, one main effect is the proficiency of some spontaneous metabolites of several PAHs to alter the cellular proteins and DNA. Biological disturbances, along with cell harm incidences, lead to metamorphoses, hormonal abnormalities, and cancer [35]. Testimony specifies that admixtures of PAHs are carcinogenic to humans. The evidence appears to occur predominantly from occupational investigations for personnel subjected to combinations including PAHs. Long-term surveys have revealed an enhanced risk of skin, lung, bladder, and gastrointestinal cancers. Yet, it is not obvious from such findings whether those people have been subjected to other carcinogenic factors in addition to aromatic compounds [32]. Other studies on animals revealed that different levels of several PAHs across long intervals lead to acquired lung cancer [36]. For the moment, some PAH-rich combinations are additionally categorized as carcinogenic to humans [37]. 

This study aimed to examine PAH impact on human health risks both before and throughout the COVID-19 pandemic.

## 2. Materials and Methods

### 2.1. Air Sampling Protocol

Makkah city is situated at latitude 21.35 [21°21′1″ N] and longitude 39.97 [39°58′1″ E]. Like most Saudi Arabian cities, the climate is very warm to hot all year round. However, the weather is particularly hot during the summer months. Twenty-four-hour PM10 samples were taken at six sample locations over Makkah city (Al-Haram, Aziziyah, Arafat, Muzdalifah, Al Nuzhah, and Al Awali), demonstrating diverse sampling locations (Table 1). This study was completed over the course of one year, from March 2020 to February 2021.

Sampling was set for one year, following the seasons: set 1, in spring, lasted 9 weeks; set 2 (summer), 10 weeks; set 3 (autumn), 8 weeks; and set 4 lasted for 10 weeks in the winter. The Hajj season for 2020–2021 was a very special event, taking place from 28 July to 2 August. That Hajj season was extraordinary owing to the exceptional global health situation triggered by the coronavirus pandemic; severe protective actions were implemented to ensure a safe, healthy Hajj for all pilgrims. Only around 1000 pilgrims attended the Hajj that year due to new crowd management regulatory limitations implemented by Saudi Arabia. The holy places in the cities of Makkah and Medina would usually host more than 2 million individuals throughout the pilgrimage period. For the first time in years, worldwide travelers were banned from the Hajj. About 70% of the participants this year were extraneous inhabitants of Saudi Arabia, with the rest being Saudi nationals. 

#### 2.1.1. Meteorological Parameters

On-site measurements using the Davis Instruments Vantage Pro Plus (Davis Instruments, Hayward, California USA) comprised temperature, wind parameters, and ultraviolet (UV) intensity. Barometric data were evaluated at a height of 10 m for 24 h and collected once a week, conferring to the USEPA set method (Method 29/2000).

#### 2.1.2. Polyaromatic Hydrocarbon (PAH) Air Sampling

A mini volume sampler (Airmetrics, Springfield, IL, USA) was used for a 24 h PM_10_ air sampling throughout all the seasons of one year, including the Hajj pilgrimage. The air sampler was located at a 10 m altitude with a 16.6 l/min flow rate, placed on a 47 mm Teflon filter designed for 24 h and collected once per week for 37 weeks in accordance with the USEPA standard method (Method 29/2000). Microbalance (CITIZEN, Tokyo, Japan) was used for filter weighing, pre- and post-sampling. Samples were stored in a dried warden under adjusted conditions of 35–40 °C with 60–70% humidity.

### 2.2. Laboratory Analysis Methods

Collected filters were removed, dehydrated, and reweighed to find the PM_10_ concentrations and stored in the dark till ready for analysis [38]. PAHs were analyzed using Perkin Elmer GC/ MS Model Clarus 600 and isolated with 10 mL of DCM/n-hexane (1:1), fractionated by column chromatography, and eluted with 20 mL of n-hexane/dichloromethane (1:1, v:v) [39]. A 2 μL sample of the extract was inserted into GC-Mass Clarus 600. The Gas Chromatography was adjusted with a diluted standard solution of 16 different PAH compounds (Supelco, Inc., Bellefonte, PA, USA), including naphthalene (NAP); dibenzo (a,h)anthracene (DBA); benzo (b)fluoranthene (BbF); acenaphthylene (ACY); acenaphthene (ACE); phenanthrene (PHE); fluorine (FLU); anthracene (ANC); fluoranthene (FLA); pyrene (PYR); benzo(a)anthracene (BaA); indeno (1,2,3-c,d)pyrene (IND); chrysene (CRY); benzo (k)fluoranthene (BkF); and benzo (ghi) perylene (BGP). These compounds were monitored in a previous study performed in Makkah city in 2013–2014 [40].

#### 2.2.1. Quality Control Measures

To ensure reliability, reproducibility, and linearity for every analysis, this study was accomplished independently by repeatedly examining a control sample. A linear calibration curve was created using blank and five-point calibrators with concentrations of 0.01, 0.1, 0.02, 0.05, and 1.0 ppm for standards compounds. 

#### 2.2.2. Statistical Analysis

One-way ANOVA (SPSS 2017, IBM, Armonk, NY, USA) was applied to assess the change in PAH intensities throughout the seasons, and the significance level was placed at *p* < 0.05.

## 3. Results and Discussion

### 3.1. PM_10_ levels and Seasonal Variations

**Figure 1:**
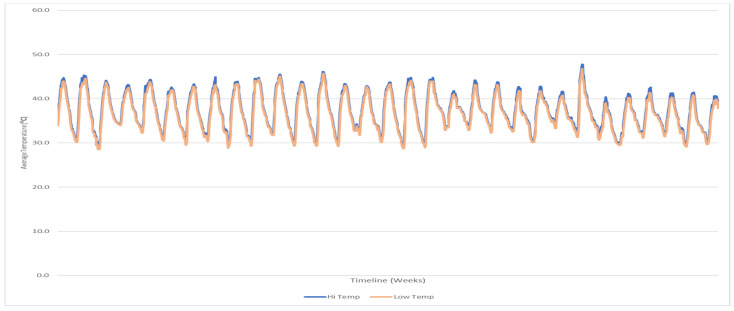
Average temperature (showing high and low temperatures) as time series over Makkah for one year (2020–2021).

**Figure 2:**
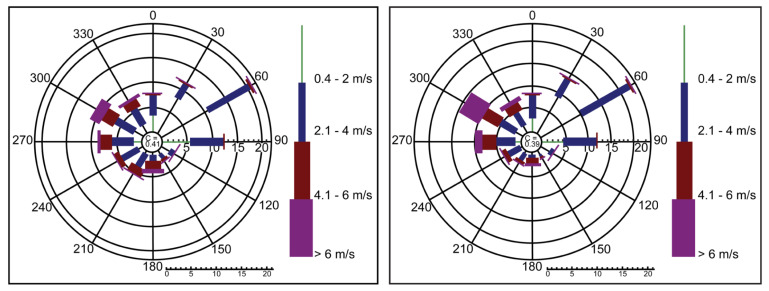
Wind changes for the Makkah region during winter and summer seasons, showing wind speed in (m/s) (2020–2021).

**Figure 3:**
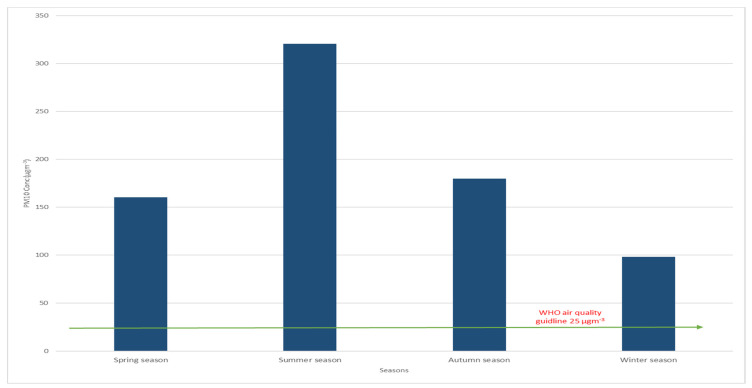
PM10 levels around Makkah throughout the seasons for one year (2020–2021). The guideline values are in accordance with the WHO statement on Ambient Air Pollution and Diseases Burden, 2018.

Table 2 shows the levels of the meteorological parameters measured and the average concentrations for 24 h PM_10_ throughout the study. The average concentrations of 24 h PM_10_ showed substantial variability in all seasons, exceeding the WHO standards for daily average exposure (25.0 µgm^−3^). The PM_10_ average concentration for set 1 (spring) was 160.3 ± 30.4 µgm^−3^, set 2 (summer) was 320.4 ± 50.4 µgm^−3^, while for sets 3 and 4 (autumn and winter), the concentrations were 179.8 ± 40.72 and 89 ± 62.7 µgm^−3^, respectively. Makkah’s weather is characterized by its dryness, which has a significant effect on the air movement in all seasons, as shown in Figure 1 and Figure 2. Consequently, the elevated concentrations of PM_10_ during the spring and summer seasons can be attributed to the reduction in air scattering during that cycle because of the reduced atmospheric boundary level. Figure 3 displays the concentrations of PM_10_ all around Makkah’s different sites compared to the WHO regulations.

### 3.2. PAHs and Seasons Changes 

**Figure 4:**
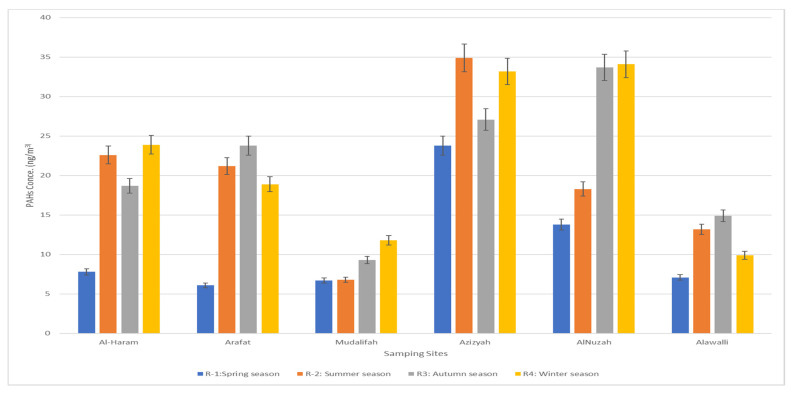
Total PAH concentrations in fractions of PM10 around Makkah in one year (2020–2021).

The PAH concentrations fluctuated from 7.67 to 34.7 ng/m^3^ in a suburban area near rush-hour traffic flow and from 6.34 to 37.4 ng/m^3^ near to profitable and light manufacturing areas, as presented in Figure 4. Earlier research conducted in the Makkah region showed that road traffic was the main source of PAHs [40]. 

**Figure 5:**
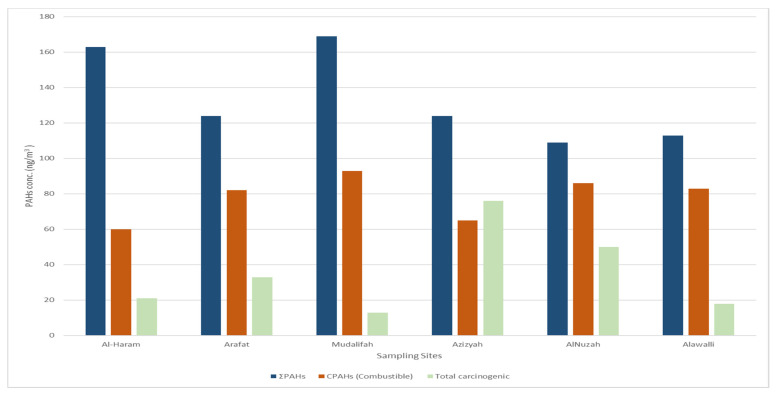
Total PAH concentrations, total carcinogenic compounds in Makkah in one year (2020–2021). Total carcinogenic compounds measured in the study included BAA, BBF, BAP, DBA, and IND, while combustible PAH compounds measured included ACY, ANC, BGP, FLA, PYR, and FLU.

The analytical ratios of PAH concentrations were analyzed in factors of PM10 filters collected as weekly concentrations. The proportions implied a larger impact of the traffic flow resources on the ambient air levels in Al Nuzah and Al-Haram. The analytical percentages of IND/BGP represent an average of five whole dust particles at all the sample locations, suggesting that the primary source of PAHs is diesel vehicles. BGP/IND proportions were about 0.83; these ratios displayed a high percentage of petrol and diesel engines in all sampling sites. These results were slightly higher than the results previously reported in China (0.28–0.75), which may be due to different site characteristics and traffic loads in China and Saudi Arabia’s sampling sites. Moreover, this displayed the high percentage of petrol and diesel engines with a high oil combustion rate at all sampling sites [40,41]. The concluded proportions of BAA/(BAA + CRY), which are more significant than the presence of diesel engines and limited industrial areas, varied between 0.5 and 1.0 at all testing sites. These results were close to the relative amounts that were calculated for diesel cars and vans that ranged between 0.38 and 0.64 [42], and manufacturing sites that ranged between 0.23 and 0.89 [41]. Normal burning generated PAHs, which may be revealed by CPAH varieties (flammable PAH types: FLT, PYR, BAA, CRY, BBF, BAP, BGP, and IND). The CPAH/ΣPAH ratios ranged between 0.54 and 0.64. Our study findings concur with those obtained for non-catalyst vehicles, heavy trucks, and buses [43]. BAA, BBF, BAP, DBA, and IND are most likely to be thought of as human carcinogens, although ACY, ANC, BGP, FLA, PYR, and FLU are uncategorized as agents of cancer risk [44]. As shown in Figure 5, the higher levels of total carcinogenic compounds were shown in the Al-Azizia, Al Muzdalifah, and Al Nuzah areas because of the high traffic density, and the lowest concentrations were found in the Al-Haram and Arafat areas throughout the year. This was the result of the COVID-19 pandemic health precautions that were implemented by the Saudi Arabian government, involving border entry limits, limitations of the Umrah and Hajj seasons, and the quarantine and social distancing that were implemented in Makkah between February 2020 and August 2021. These measures correlated with the lowered spread of COVID-19 [45] and directly related to decreasing PM10 levels to an annual average of 188 µgm^−3^, consequently lowering the PAH concentrations analysed throughout this exceptional year. Our findings strongly agree with a previous study conducted in Makkah during 2014, which showed an annual average PM10 concentration of 340 µgm^−3^ [38]. 

Our study showed that social distancing measures have highly influenced both the air contamination in the Makkah region and people’s behaviours. Changes in people’s behaviours from February to June 2020 to comply with government restrictions and recommendations had a great effect on reducing the transmission of COVID-19 [46,47], as well as ambient air pollution and PAH concentrations, in all sampling locations. This observation indicates that consistent measures should have affected COVID-19 transmission in the community and air quality in Makkah.

## 4. Conclusions

Ambient air quality, with its chemical components, particularly polyaromatic hydrocarbon compounds that contain certain carcinogenic compounds, is considered a significant problem in Makkah. PM10 concentrations over one year, representing numerous anthropogenetic occupations in the Makkah area, comprising domestic areas, a combination of suburban and light industrial areas, and Hajj areas, were all above the standard rates of the Saudi Presidency of Meteorological and Environment (35 µgm^−3^) and the World Health Organization’s guidelines (25 µgm^−3^) on PAH levels in ambient air. The study period is considered extraordinary, as the Saudi Arabian government had undertaken successful precautionary procedures during the COVID-19 pandemic, including such self-imposed measures as social distancing and remote working and learning, which all had a great effect on the pandemic spread and in reducing air pollution in Makkah. More studies are required to examine PAHs and their carcinogenic effects related to short- and long-term exposure after the pandemic measures are eased across Makkah.

## Figures and Tables

**Table 1:** Sampling location basic information and site pollution attributes.

Sampling Locations	Location	Sampling Site Features
**Al-Haram**	21°25′19.2″ N,39°49′33.6″ E	Intense traffic flow due to Hajj and Umrah seasons all year around
**Arafat**	About 20 km from Central Makkah	Intense transportation of trucks and buses based on Hajj season
**Muzdalifah**	21°24′33″ N, 39°54′11″ E	Intense transportation of trucks and buses based on Hajj season
**Aziziyah**	32°31′48″ N 1,3°0′36″ E	Domestic region with moderate traffic flow capacity excluding Hajj periods
**Al Nuzhah**	Nearly 5 km away from Al-Haram	Domestic, small business district with moderate to high-level commuter transportation capacity
**Al Awali**	21°19′29″ N, 39°53′24″ E	Rural location has minimal transportation with small business-related activities

**Table 2:** Some meteorological parameters along with PM10 levels in Makkah throughout one year (2020–2021).

Measurements	Set 1 9 Weeks (Spring)	Set 2 10 Weeks (Summer)	Set 3 8 Weeks (Autumn)	Set 4 10 Weeks (Winter)
Temperature °C	30.4 ± 1.56	39.5 ± 3.25	35.6 ± 1.88	29.1 ± 1.47
Wind Speed (ms^−1^)	6.5 ± 3.2	5.0 ± 1.3	6.6 ± 1.8	4.3 ± 1.7
PM10 (µgm^−3^)	120.1 ± 52.2	223.4 ± 30.4	77.6 ± 36.72	89 ± 62.7

## Data Availability

The data presented in this study are available on request from the corresponding author.
